# Differential effects of size-specific particulate matter on the number of visits to outpatient fever clinics: A time-series analysis in Zhuhai, China

**DOI:** 10.3389/fpubh.2022.972818

**Published:** 2022-12-23

**Authors:** Duo Li, Rui He, Peixin Liu, Hong Jiang

**Affiliations:** ^1^Department of Operations, Zhuhai People's Hospital, Zhuhai, China; ^2^Universitat Pompeu Fabra, Barcelona, Spain; ^3^Department of Spine and Bone Disease, Zhuhai People's Hospital, Zhuhai, China; ^4^Faculty of Medicine, Macau University of Science and Technology, Macau SAR, China

**Keywords:** PM_2.5_, PM_10_, coarse particulate matter, fever clinic, outpatient visits

## Abstract

**Introduction:**

While many studies have investigated the adverse effects of particulate matter (PM), few of them distinguished the different effects of PM_2.5_, PM_10_, and coarse PM (PMc) on outpatients with fever. Our study aimed to estimate and compare the acute cumulative effects of exposure to three size-specific particles on the number of visits to outpatient fever clinics.

**Methods:**

To examine the association between daily PM concentrations and outpatients in fever clinics, a generalized additive Poisson model was applied, stratified by sex, age, and season.

**Results:**

Our study included 56,144 outpatient visits in Zhuhai, from January 2020 to June 2021. On the current day, each 10 mg/m^3^ increment of PM_10_ and PMc were estimated to increase fever clinic visits by 1.74% (95% CI: 0.59%, 2.91%) and 4.42 % (2.30%, 6.58%), respectively. Cumulative effects enhanced from lag01 to lag05 for PM_10_ and PMc, and PMc had the strongest impact [ER = 8.92% (5.91%, 12.01%) at lag05]. Female outpatients and outpatients aged 14 years and above had an increased PM-related risk. During the cold season, significant effects could be observed for the three-size PM, while only PMc showed the impact during the warm season.

**Discussion:**

Overall, the three size-specific PM exerted different effects on the fever clinic visits. Strategies to control the concentrations of PM are still necessary, especially against PM_10_ and PMc.

## 1. Introduction

Particulate matter (PM) is a mixture of particles suspended in air varying in size and composition. According to the World Health Organization, PM was divided into PM_10_ (inhalable particulate matter with an aerodynamic diameter of <10 μm) and PM_2.5_ (fine particulate matter with an aerodynamic diameter of <2.5 μm) (1). Particles with an aerodynamic diameter between 2.5 and 10μm are defined as coarse particulate matter (PMc). Exposure to PM has been related to various adverse health effects (2–5).

Particulate matter was identified to be associated with increased mortality of respiratory diseases in previous studies. A study that included 652 cities in 24 countries across the globe showed that PM_2.5_ and PM_10_ increased daily respiratory mortality significantly (6). There were studies focused on the morbidity of respiratory diseases related to PM. A meta-analysis conducted in 2020 revealed that PM increased pneumonia-specific hospital admissions and emergency room visits, respectively (7). We found less research exploring the relationship between PMc and respiratory hospital admissions, and findings remained inconsistent. For example, a study conducted in California observed significant associations between respiratory emergency department visits and coarse particle levels, while PMc found no effects on pneumonia and acute respiratory infection (8). Another study from Toronto suggested the impact of PMc on respiratory infections in children (9). During the COVID-19 pandemic, exposure to PM was proven to be positively correlated with the spread of COVID-19 in recent studies (10).

In China, fever clinics serve as the first line of defense against infectious diseases, playing an important role in screening outpatients with fevers in public hospitals, especially during the COVID-19 pandemic. Before the establishment of fever clinics, outpatients with fever usually visited the emergency departments. In Zhuhai People's Hospital, outpatients with a body temperature of 37.3°C or higher were required to visit the fever clinic first (11). Clinically, the main cause of fever is infection, which was mostly accompanied by respiratory symptoms in our fever clinics.

Chinese researchers have been exploring the impacts of particulate matter on respiratory morbidity. Studies from several Chinese cities indicated that respiratory outpatients increased by PM concentration (5, 12). Some studies also identified the potential impacts on hospital visits by vulnerable populations such as children (13). However, as the establishment of fever clinics in China is unique worldwide, very little is known about the association between size-specific PM and fever clinic visits in China. Studies aiming to compare the effects of size-specific PM on outpatients with fever are still limited. This study aimed to identify and differentiate the acute and cumulative effects of exposure to PM_2.5_, PM_10_, and PMc on the number of outpatient visits to fever clinics. This could provide a scientific basis for air pollution control and fever clinic management.

## 2. Materials and methods

### 2.1. Study settings

This study was conducted in Zhuhai, a core city on the west coast of the Pearl River Delta in China. It is an important part of the Guangdong-Hong Kong-Macao Greater Bay Area. Zhuhai lies between 21.48°N−22.27°N latitude and 113.03°E−114.19°E longitude, with subtropical and transitional tropical marine climate. In the city, the climate is warm and humid throughout the year, while winters are relatively sunny and dry. Zhuhai was rated as the top 10 cities in China with the cleanest environment (14). It has 2.5 million people in 1736.45 square kilometers (km^2^) in 2021 (15). This study was approved by the Ethics Committee in Zhuhai People's Hospital [No: LW-(2022) No. 9].

### 2.2. Data on fever clinic visits

Outpatient records were extracted from the information system of Zhuhai People's Hospital in Zhuhai from 18 January 2020 to 28 June 2021. Located in the City Center of Zhuhai, the hospital accepts most outpatients downtown. The number of daily outpatient fever clinic visits was calculated as a sum of the entire day. Recorded information includes age, sex, date of hospital visits, and clinical diagnosis.

### 2.3. Air quality and meteorological data

The hourly concentration of air pollution was collected from the air quality sharing platform administered by China's Ministry of Environmental Protection. We calculated daily data from the hourly data concerning concentrations of PM_10_, PM_2.5_, ozone (O_3_), nitrogen dioxide (NO_2_), sulfur dioxide (SO_2_), and 8-hour maximum levels of O_3_. Since the data on PMc concentrations were not available, we obtained the data by subtracting PM_10_ and PM_2.5_. In addition, we collected daily meteorological data including daily mean temperature (°C) and relative humidity (%) from the National Weather Data Sharing System of China.

### 2.4. Statistical analysis

We applied a time-series analysis to investigate the effects of PM_10_, PM_2.5_, and PMc, respectively. Given that daily outpatient visits followed a Quasi-Poisson distribution according to previous research, an over-dispersed generalized additive model (GAM) was applied (16, 17). The model adjusted mean temperature, relative humidity, calendar time, day of the week (DOW), and public holiday (PH). Previous studies and the Akaike information criterion (AIC) were both considered in deciding the degrees of freedom. The model is determined as follows:


$$\begin{array}{l} \log\left(E\left(Yt\right)\right)=\beta^{*}Zt+ns\left(time,df=\frac{7}{year}\right)\\ +ns\left(temp,df=4\right)+ns\left(relative\;humidity,df=4\right)\\ +factor\left(DOW\right)+factor\left(PH\right)+\;\alpha \end{array}$$

Where, *E(Yt)* refers to the expected outpatient visits for fever clinic on day t, *Zt* refers to mean PM concentration on day t, time refers to calendar time, which was used to control unmeasured long-term trend, and temp refers to the average temperature on the current day, relative humidity refers to relative humidity on the current day, DOW, PH are dummy variables, β refers to the coefficient for *Zt*, ns refers to a natural cubic smooth function, df refers to the degree of freedom, and α refers to the intercept. The relative risk is calculated by taking the logarithm of β.

In the main model, we considered the effects of single lags days (lag0–lag5) and cumulative effects (lag01–lag05). The effects were measured by excess risk (ER) with a corresponding 95% confidence interval (95% CI). ER was calculated as follows, indicating the percentage change in the number of outpatients in fever clinics with each per unit increase in PM_2.5_, PM_10_, and PMc.


$$\begin{array}{l} Excess\;Risk=\left(Relative\;Risk-1\right)\times 100\% \end{array}$$

We plotted an exposure–response curve to show the association between PM and outpatients in fever clinics. Due to the potential effect of modifications, stratified analysis was conducted by age, sex, and season. Consistent with the existing literature, the warm season was defined as April to September, and the cold season was defined as October to March (18). The 95% CI was used to test the significant differences between subgroups. To examine the robustness of our results, sensitivity analysis was conducted by performing two-pollutant models and adding air pollutants including SO_2_, NO_2_, and O_3_ in the model, respectively.

The analysis was conducted by using R software (version 4.1.1) with packages “mgcv” and “tsModel.” Statistical significance was considered when a two-sided *p*-value was <0.05.

## 3. Results

From 18 January 2020 to 28 June 2021, a total of 56,144 fever clinic visits were recorded in our hospital (Table 1). Patients older than 14 years accounted for 45.2% of the outpatients and 54.7 % of outpatients in the fever clinic were male patients. The study was conducted on more warm days (71.7%) than on cold days. The daily mean (SD) concentrations of PM_10_, PM_2.5_, PMc, SO_2_, NO_2_, and O_3_ were 35.7 (23.5) μg/m^3^, 19.3 (12.6) μg/m^3^, 16.4 (12.1) μg/m^3^, 53.1 (40.6) μg/m^3^, 23.2 (15.0) μg/m^3^, and 85.1 (38.3) μg/m^3^, respectively, in Zhuhai, China. The daily mean temperature was 23.6°C, and the relative humidity was 78.2% (Table 2). The new air quality guidelines by the World Health Organization set standards for the daily mean of PM_2.5_, PMc, and PM_10_ as 15 μg/m^3^, 30 μg/m^3^, and 45 μg/m^3^, respectively (1). The average concentrations of PM_10_ and PMc in Zhuhai reached the standard, while that of PM_2.5_ did not.

**Table 1:** Demographic characteristics of outpatients in fever clinics in Zhuhai, 2020–2021.

	**Overall (*****N*** = **56144)**	
	***n***	**(%)**
**Age**		
< 14	30702	54.68%
≥14	25442	45.32%
**Sex**		
Female	25402	45.24%
Male	30742	54.76%
**Season**		
Cold	24789	44.15%
Warm	31355	55.85%

**Table 2:** Summary statistics of meteorological factors and daily air pollution in Zhuhai, 2020–2021.

	**Mean**	**SD**	**Median**	**P_25_**	**P_75_**	**Minimum**	**Maximum**
Mean temperature	23.6	5.2	24.0	19.5	28.0	7.0	31.5
Relative humidity (%)	78.2	11.8	80.0	74.0	85.9	27.9	98.0
PM_10_ (μg/m^3^)	35.7	23.5	31.0	17.0	47.0	5.0	136.0
PM_2.5_ (μg/m^3^)	19.3	12.6	17.0	9.0	26.0	3.0	70.0
PMc (μg/m^3^)	16.4	12.1	13.0	7.0	22.0	1.0	77.0
SO_2_ (μg/m^3^)	53.1	40.6	39.2	24.8	70.3	4.6	272.0
NO_2_ (μg/m^3^)	23.2	15.0	20.0	12.0	31.0	4.0	95.0
O_3_ (μg/m^3^)	85.1	38.3	78.0	53.0	108.2	18.0	226.0

The correlations among the key pollutants are shown in Supplementary Table S1. Criteria of correlation coefficients were set following a review of previous studies (low for |r| < 0.4; moderate for 0.4≤ |r| ≤0.6; high for |r| > 0.6). High correlations were observed between the three-size particles (*r* = 0.95 between PM_2.5_ and PM_10_, r = 0.95 between PM_2.5_ and PMc, r = 0.81 between PMc and PM_10_). NO_2_ was also highly correlated with PM_2.5_, PM_10_, and PMc, respectively (*r* = 0.77, 0.78, and 0.71).

**Figure 1:**
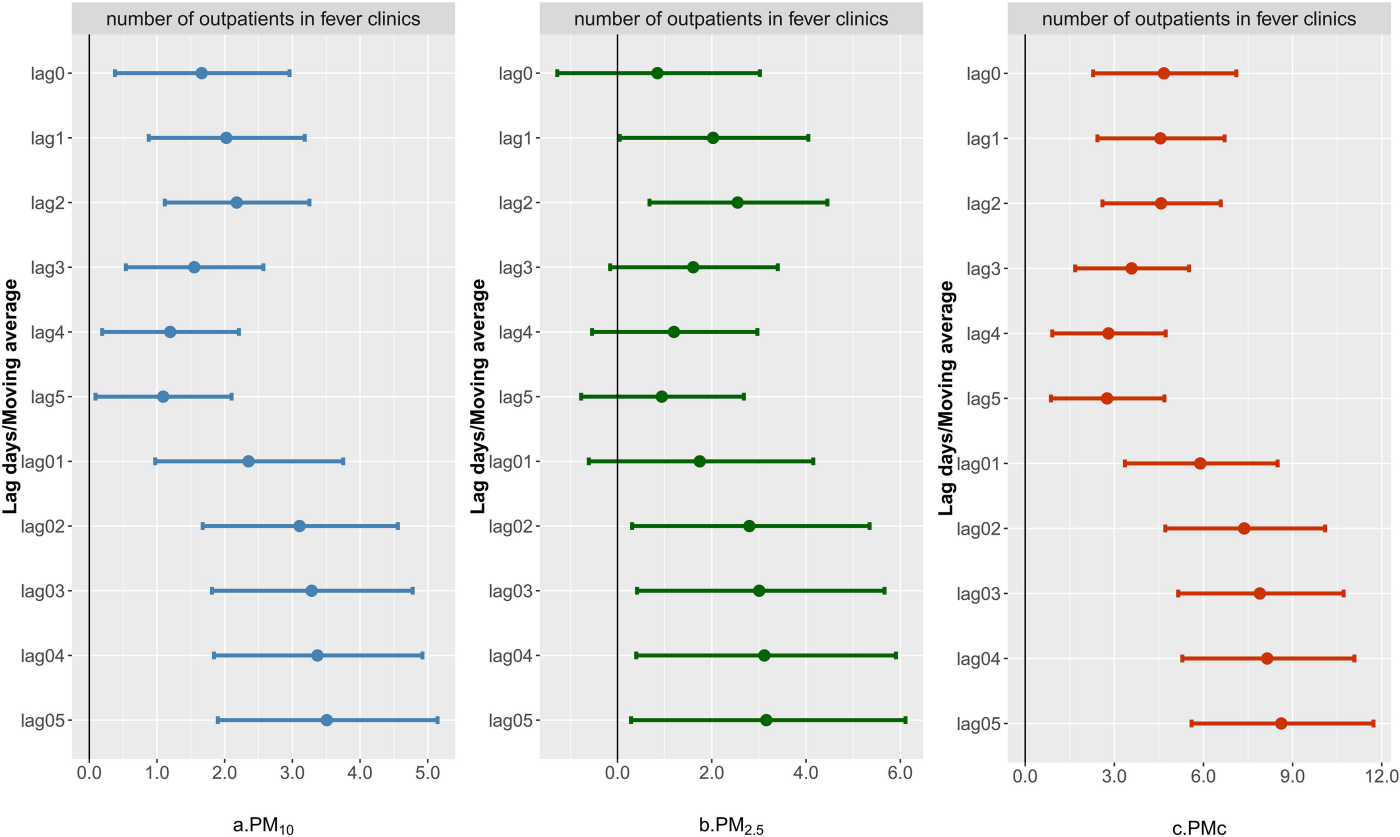
Excess risk of number of outpatient visits to fever clinics for each 10 μ*g*/*m*^3^ increase in PM_10_, PM_2.5_, and PMc. The ER and corresponding 95% CI were derived from an over-dispersed generalized additive model, with calendar time, weather conditions, day of the week (DOW), and public holiday (PH) controlled.

In Figure 1, the associations of PM_10_, PM_2.5_, and PMc with the number of fever clinic visits were displayed. The size-specific PM showed acute effects at lag0, and different results of ER could be observed. PM_10_ and PMc were significantly associated with the number of fever clinic visits [ER = 1.74% (0.59%, 2.91%) for PM_10_, ER = 4.42 % (2.30%, 6.58%) for PMc], while PM_2.5_ showed no significant effect on the current day [ER = 1.34% (−0.68%, 3.40%)]. For PM_2.5_, effects of single lag days appeared since lag1 and disappeared from lag3, while elevated effects could be seen for PM_10_ and PMc from lag1 to lag5. As for the cumulative effects, ER increased from lag01 to lag05 for PM_10_ and PMc, respectively, but to a different extent. PM_2.5_ was insignificant at lag01, but the risk appeared and lasted from lag03 to lag05. PMc had the strongest effects [ER = 8.92% (5.91%, 12.01%) at lag05] compared with PM_2.5_ and PM_10_.

**Figure 2:**
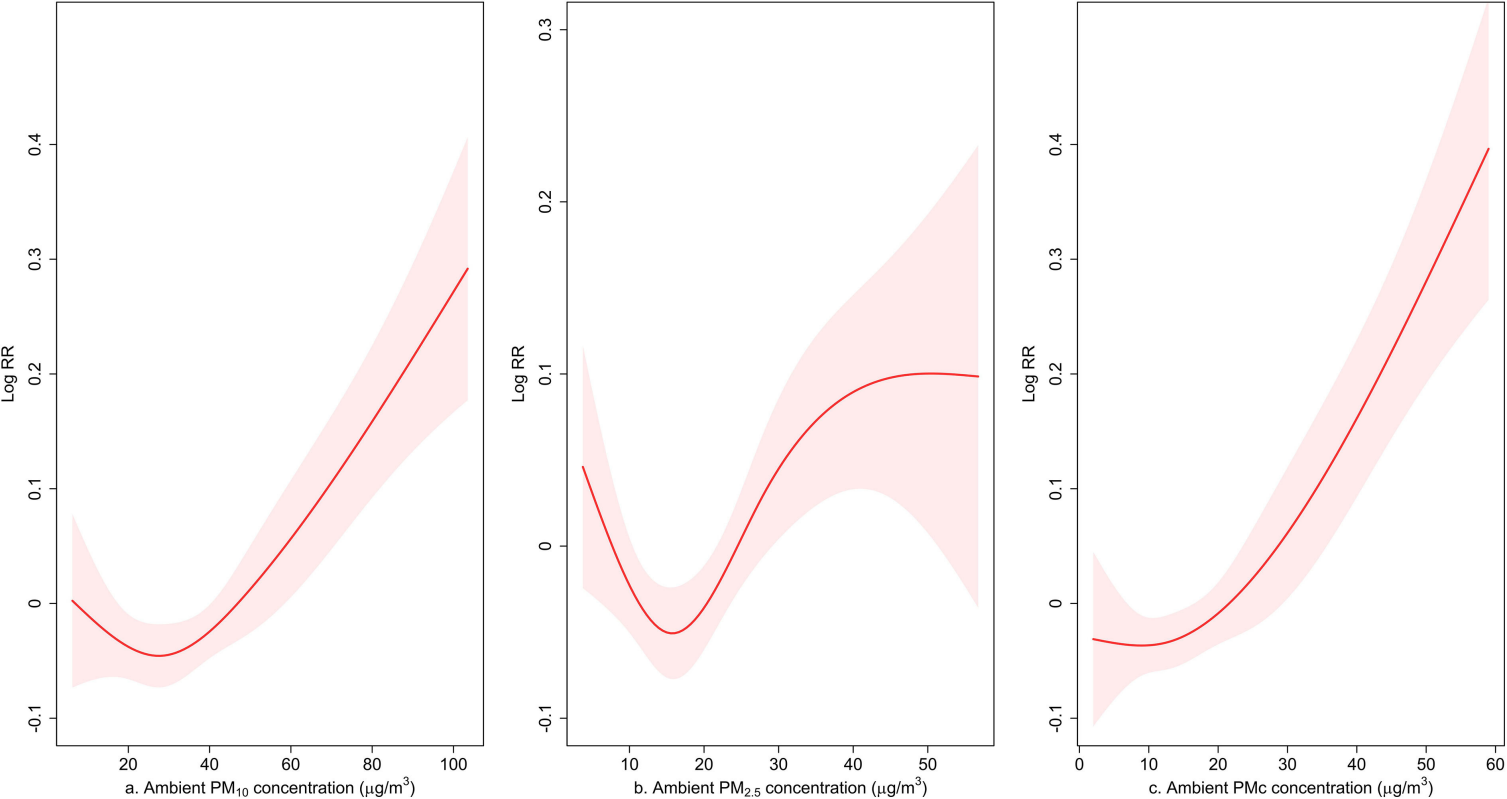
Exposure–response curve for the association between PM_10_, PM_2.5_, and PMc (lag03) and the number of outpatient visits to fever clinics. The line represents the point estimates and the shading indicates corresponding 95% CIs, which were derived from an over-dispersed generalized additive model, with weather conditions, day of the week (DOW), and public holiday (PH) controlled.

Figure 2 shows the exposure–response curves between each 10 μg/m^3^ increase of PMs (lag03) and the number of fever clinic visits. For PM_10_ and PMc, an approximately linear relationship could be observed from 20μg/m^3^ and above. Generally, outpatients in the fever clinic increased with the ambient concentration. Though the curve for PM_2.5_ was non-linear, we could still observe a linear relationship from 20 μg/m^3^ to 40 μg/m^3^.

A stratified analysis was conducted, and the results are shown in Table 3. We could find significant and increased risks among female patients for the three-size PM. Among male patients, however, the association between PM_2.5_ and fever clinic visits was not observed and male patients only elevated the PMc-related risk. For different age groups, we found the association between PM_2.5_ and outpatients younger than 14 was not significant. Outpatients aged 14 and above suffered a higher risk of fever clinic visits. During the cold season, significant effects could all be observed for all the three-size PM, and the strongest effect appeared at PM_2.5_ [ER = 6.69% (2.40%, 11.16%)]. Only the risk of PMc remained significant during the warm season.

**Table 3:** Excess risk (%) and 95% CI of outpatient visits to fever clinics associated with a 10 μg/m^3^ increase in PM_10_, PM_2.5_, and PMc (lag03) concentrations, stratified by sex, age, and season.

	**Excess Risk (95% CI)**		
	**PM_10_**	**PM_2.5_**	**PMc**
**Sex**			
Male	3.09 (1.48, 4.74)^*^	2.18 (−0.70, 5.15)	8.17 (5.17, 11.26) ^*^
Female	3.81 (2.14, 5.50)^*^	5.1 (2.10, 8.19)^*^	7.46 (4.35, 10.65)^*^
**Age**			
< 14 years	2.09 (0.53, 3.67)^*^	0.46 (−2.26, 3.26)	6.70 (3.78, 9.70)^*^
≥14 years	4.08 (1.99, 6.22)^*^	5.93 (2.13, 9.87)^*^	7.48 (3.62, 11.49)^*^
**Season**			
Cold	3.85 (1.47, 6.29)^*^	6.69 (2.40, 11.16)^*^	4.48 (0.53, 8.58)^*^
Warm	1.27 (−1.41, 4.03)	0.3 (−2.67, 3.37)	6.14 (1.04, 11.49)^*^

* Significant result.

To test the robustness of our results, two-pollutant models are shown in Table 4. Overall, ER for PM remained significant and even increased when adding SO_2_ into the model, while adding NO_2_ and O_3_ led to decreased results. For PM_10_, adding PM_2.5_ changed ER (lag0) from 1.74% (0.59%, 2.91%) to 5.89% (3.20%, 8.65%). When adding PM_10_ to the model of PM_2.5_, ER changed from 1.34% (−0.68%, 3.40%) to −7.36 (−11.47%, −3.06%). For PMc, adding PM_10_ and PM_2.5_ into the model both increased ER significantly.

**Table 4:** Excess risk (%) and 95% CI of outpatient visits to fever clinics associated with each 10 μg/m^3^ increase in PM_10_, PM_2.5_, and PMc (lag0, lag03) concentrations in single pollutant models and two-pollutant models, respectively.

		**Excess risk (95%CI)**	
		**lag0**	**lag03**
**PM**_**10**_		1.74 (0.59, 2.91)^*^	3.42 (1.99, 4.86)^*^
	+NO_2_	1.72 (0.38, 3.08)^*^	3.23 (1.75, 4.72)^*^
	+SO_2_	1.85 (0.69, 3.02)^*^	3.47 (2.04, 4.92)^*^
	+O_3_	1.15 (−0.25, 2.57)	3.06 (1.52, 4.62)^*^
	+PM_2.5_	5.89 (3.20, 8.65)^*^	4.32 (2.60, 6.07)^*^
	+PMc	−1.90 (−4.31, 0.57)	2.38 (0.63, 4.16)^*^
**PM**_**2.5**_		1.34 (−0.68, 3.40)	3.49 (0.92, 6.13)^*^
	+NO_2_	0.56 (−1.80, 2.97)	2.98 (0.35, 5.69)^*^
	+SO_2_	1.48 (−0.55, 3.55)	3.63 (1.05, 6.28)^*^
	+O_3_	−0.56 (−2.94, 1.87)	2.44 (−0.30, 5.24)
	+PM_10_	−7.36 (−11.47, −3.06)^*^	1.89 (−1.20, 5.08)
	+PMc	−1.90 (−4.31, 0.57)	1.44 (−1.29, 4.25)
**PMc**		4.42 (2.30, 6.58)^*^	7.90 (5.26, 10.61)^*^
	+NO_2_	4.53 (2.23, 6.89)^*^	7.56 (4.86, 10.34)^*^
	+SO_2_	4.63 (2.50, 6.80)^*^	7.87 (5.23, 10.58)^*^
	+O_3_	3.89 (1.50, 6.33)^*^	7.33 (4.55, 10.18)^*^
	+PM_10_	7.94 (3.16, 12.95)^*^	8.29 (5.04, 11.64)^*^
	+PM_2.5_	5.89 (3.20, 8.65)^*^	8.53 (5.66, 11.48)^*^

* Significant result.

## 4. Discussion

This is the first study in China that elaborated on the associations between PM_2.5_, PM_10_, PMc, and fever clinic visits. We investigated the acute and cumulative effects of exposure to the three size-specific particles on the number of outpatients in fever clinics. There was a total of 56,144 fever clinic visits from 2020 to 2021 in our hospital. On the current day (lag0), we found that the acute effects only appeared for PM_10_ and PMc, and PMc had a stronger impact. As for the cumulative effects, exposure to PM_2.5_, PM_10_, and PMc was all significantly associated with increased fever clinic visits at lag03. Overall, PMc tended to have the strongest adverse effects compared with PM_2.5_ and PM_10_. The subgroup analysis demonstrated that PM_10_ and PMc were associated with the number of outpatient fever clinic visits for both genders but with different effects. Among different age groups, we found that outpatients older than 14 years suffered significant and higher risks from exposure to PM. During the cold season, we could see robust and stronger effects for all three sizes of particles compared with the warm season.

Our findings for PM are in line with the existing knowledge about the mechanisms of particles to induce pulmonary inflammation and damage. Previous studies demonstrated that PM entering the lungs initiates an inflammatory response and a pro-inflammatory immune response (19, 20). These responses follow an oxidative stress reaction that induces epithelial cell damage and activation (21). The adverse effects underlie pathologies including infections, which are the major causes of fever (19).

Our study mainly focused on the differential effects of the three size-specific PM. For the acute effects, we found no association between PM_2.5_ and the number of outpatients in fever clinics on the current day. The result was consistent with a meta-analysis of respiratory hospital admissions by the WHO region, where COPD was excluded (22). However, there were studies in China found significant effects on respiratory hospital admissions for PM_2.5_ (23, 24). As for PM_10_ and PMc, the acute effects were both observed in our study. A cases-crossover study between two Chinese metropolitan populations found that each 10 μg/m^3^ in PM_10_ increased risks for daily emergency department visits by 1.7% (1.5%, 2.0%) (24), which was quite close to our result [1.74% (0.59%, 2.91%)]. In the current study, the risks for cumulative effects were higher than the acute effects for PM, and the strongest effect appeared at lag04 or lag05 for each size of PM. The findings varied in different studies since the strongest cumulative effects appeared on different lag days (17, 23). In another time-series study from China, researchers demonstrated that a 10 g/m^3^ increase in PMc was associated with 6.37% (95% CI 1.84, 11.10) hospital visits for respiratory diseases at lag06 (12). Compared with their findings, our study identified higher risks of PMc [ER = 8.92% (5.91%, 12.01%) at lag05]. The possible reason for the inconsistent evidence was that chemical components and source categories of PM varied in some regions. Some particles could be more toxic to humans, which may cause a stronger health impact. In the current study, PMc had the greatest impact on outpatient visits in fever clinics regardless of gender, age, and season. This result could be due to particles larger than PM_2.5_ causing increased cytokine release and a more severe inflammatory response (21). Another possible explanation is that PMc is small enough to penetrate and deposit in the respiratory tract than PM_10_ (25)_._ These may interpret why PMc would have robust and stronger effects and why cumulative exposure would show higher risks.

Previous studies have suggested that patients of younger ages should be considered to be susceptible to PM-related respiratory hospital admissions (13, 26, 27). A study conducted between two Chinese metropolitan populations also found that children younger than 14 years suffered higher PM-induced risks of emergency department visits (24). However, in our study, we demonstrated that outpatients aged 14 and above were more at risk of fever clinic visits. In our fever clinics, we divide outpatients into two groups based on their age (14 years) to provide better treatments for both children and adults. Although our result was inconsistent with some studies, it could still provide practical guidance for our fever clinic management.

In terms of gender differences, we found the risk brought by PM_10_ was higher for female outpatients, while PMc had a stronger impact on male outpatients. It was worth noting that PM_2.5_ only had a significant impact on male outpatients. A study from Lanzhou, China suggested that male patients were more sensitive to PM-related (PM_10_ and PM_2.5_) risk than female patients for respiratory disease emergency department visits (28). An animal experiment also indicated that male patients expressed the inflammatory markers differently, which may link to acute pulmonary infection (29). However, a meta-analysis on associations between short-term exposure to air pollution and respiratory hospital admissions did not observe significant gender differences (7). A possible explanation for the inconsistent findings was that personal PM exposure levels for male and female patients varied by occupation and lifestyle in different regions. Further exploration is needed to identify gender differences clearly in future studies.

In our study, we conducted a population-based time-series study to differentiate the acute and cumulative effects of PM on fever clinic outpatient visits. Despite the strengths of our study, there are some limitations. First, like many studies that explored the effects of air pollution on diseases, ecological bias is inevitable. Second, the data on PMc concentrations were obtained by subtracting PM_2.5_ and PM_10_, which may cause misclassification of the exposure of PMc. Third, only one hospital in Zhuhai was included in this study, and the representativeness of results may be affected. Finally, the two-pollutant models were performed to assess the robustness of our results. However, air pollutants were moderately or highly correlated, which might add uncertainty to our main results (30). Further studies to measure correlated air pollutants are needed.

## 5. Conclusion

Our study identified differential effects of PM_2.5_, PM_10_, and PMc on fever clinic visits in terms of the effect magnitude, effect stability, and susceptible population. The acute effects only appeared for PM_10_ and PMc, while the cumulative effects could be observed for PM_2.5_, PM_10_, and PMc. PMc tended to have the strongest adverse effects compared with PM_2.5_ and PM_10_. Outpatients aged 14 and above suffered a higher risk of fever clinic visits. Male patients only had elevated PMc-related risk, and female patients had increased risk for all the three-size specific PM. We could see robust and stronger adverse effects of the three-size PM during the cold season. These findings added to the existing knowledge of the adverse health effects of PM. Strategies to control the concentrations of PM are still necessary, especially against PM_10_ and PMc.

## Data availability statement

The original contributions presented in the study are included in the article/Supplementary material, further inquiries can be directed to the corresponding author.

## Ethics statement

The studies involving human participants were reviewed and approved by the Ethics Committee in Zhuhai People's Hospital. Written informed consent for participation was not provided by the participants' legal guardians/next of kin because: Data in our study was desensitized before we obtained. Therefore, exemption of informed consent was proved.

## Author contributions

DL designed the study and wrote the manuscript. RH and PL revised the manuscript. HJ designed the study, collected the data, revised the manuscript, and provided comments on the final draft of the manuscript. All authors contributed to the article and approved the submitted version.
