# Supplementary material for: Differential effects of size-specific particulate matter on the number of visits to outpatient fever clinics: A time-series analysis in Zhuhai, China
Source: Front Public Health. 2022 Dec 23;10:972818. doi: 10.3389/fpubh.2022.972818 (PMC9816473; doi:10.3389/fpubh.2022.972818)
Supplement: Supplementary file 2 [file Data_Sheet_2.docx]

**Table S 1** Spearman correlation between air pollutants in Zhuhai,2020-2021

|  | **PM_2.5_** | **PM_10_** | **PMc** | **SO_2_** | **NO_2_** | **O_3_** |
| --- | --- | --- | --- | --- | --- | --- |
| **PM_2.5_** | 1 | 0.95 | 0.81 | -0.05 | 0.77 | 0.57 |
| **PM_10_** | 0.95 | 1 | 0.95 | -0.01 | 0.78 | 0.57 |
| **PMc** | 0.81 | 0.95 | 1 | 0.03 | 0.71 | 0.5 |
| **SO_2_** | -0.05 | -0.01 | 0.03 | 1 | -0.07 | -0.19 |
| **NO_2_** | 0.77 | 0.78 | 0.71 | -0.07 | 1 | 0.27 |
| **O_3_** | 0.57 | 0.57 | 0.5 | -0.19 | 0.27 | 1 |

**Table S 2** Exces risk(%) and 95%CI of number of outpatient visits in fever clinics.

|  | **PM_2.5_** | **PM_10_** | **PMc** |
| --- | --- | --- | --- |
| lag0 | 1.34 (-0.68, 3.40) | 1.74 (0.59, 2.91)* | 4.42 (2.30, 6.58)* |
| lag1 | 2.23 (0.28, 4.22)* | 2.06 (0.95, 3.18)* | 4.50 (2.46, 6.59)* |
| lag2 | 2.79 (0.91, 4.71)* | 2.30 (1.23, 3.38)* | 4.72 (2.75, 6.73)* |
| lag3 | 1.66 (-0.11, 3.48) | 1.62 (0.60, 2.65)* | 3.74 (1.83, 5.70)* |
| lag4 | 1.10 (-0.64, 2.87) | 1.17 (0.17, 2.19)* | 2.85 (0.95, 4.79)* |
| lag5 | 0.73 (-0.99, 2.48) | 0.98 (-0.02, 1.99) | 2.60 (0.71, 4.52)* |
| lag01 | 2.21 (-0.04, 4.52) | 2.39 (1.13, 3.67)* | 5.62 (3.31, 7.99)* |
| lag02 | 3.24 (0.81, 5.72)* | 3.14 (1.79, 4.50)* | 7.09 (4.60, 9.64)* |
| lag03 | 3.49 (0.92, 6.13)* | 3.44 (2.01, 4.88)* | 7.90 (5.26, 10.61)* |
| lag04 | 3.55 (0.83, 6.35)* | 3.58 (2.07, 5.11)* | 8.36 (5.55, 11.24)* |
| lag05 | 3.48 (0.59, 6.45)* | 3.70 (2.10, 5.34)* | 8.92 (5.91, 12.01)* |
